# Species Composition and Emergence Patterns of *Agriotes litigiosus*, *A. brevis* and *A. sordidus* (Coleoptera: Elateridae) in Central Italy

**DOI:** 10.3390/insects17020172

**Published:** 2026-02-05

**Authors:** Abdalhadi M.A. Abulebda, Giorgio Sperandio, Sara Ruschioni, Matteo Pacella, Maria Chiara Battistelli, Nunzio Isidoro, Paola Riolo

**Affiliations:** Department of Agricultural, Food and Environmental Sciences, Polytechnic University of Marche, Via Brecce Bianche, 60131 Ancona, Italy; a.abulebda@pm.univpm.it (A.M.A.); g.sperandio@staff.univpm.it (G.S.); m.pacella@staff.univpm.it (M.P.); m.c.battistelli@pm.univpm.it (M.C.B.); n.isidoro@staff.univpm.it (N.I.); p.riolo@staff.univpm.it (P.R.)

**Keywords:** *Agriotes*, click beetles, wireworms, IPM, population dynamics

## Abstract

Click beetles (*Agriotes* spp.) are major agricultural pests whose larvae, known as wireworms, cause serious damage to a wide range of crops. Effective management requires a clear understanding of species composition and adult emergence patterns. This study investigated three *Agriotes* species in central Italy, focusing on their abundance and phenology in the Marche region. Field monitoring was conducted at six sites using pheromone traps from 2024 to 2025. Three species were detected: *A. litigiosus*, *A. brevis*, and *A. sordidus*, with *A. litigiosus* being the most abundant. Adult emergence displayed species-specific timing, occurring earliest in *A. brevis*, followed by *A. sordidus* and subsequently *A. litigiosus*. These findings highlight the importance of species-specific monitoring to optimize pest-control strategies. A deeper understanding of *Agriotes*’ flight dynamics can support more effective and sustainable integrated pest management (IPM) strategies, reducing reliance on chemical control and minimizing crop damage.

## Simple Summary

Click beetles (*Agriotes* spp.), whose larvae are known as wireworms, are among the harmful pests of European and North American crops, feeding on seeds and young roots. We monitored adult populations of three species (*A. litigiosus*, *A. brevis* and *A. sordidus*) in central Italy using pheromone traps at six sites. *Agriotes litigiosus* was the most common, followed by *A. sordidus* and *A. brevis*. The species emerged in sequence, with *A. brevis* emerging first, then *A. sordidus*, and finally *A. litigiosus*. Understanding when these beetles are most active can help farmers apply more precise and sustainable pest-control methods, reducing crop losses and limiting the use of chemical insecticides.

## 1. Introduction

Click beetles (Coleoptera: Elateridae), whose larvae are known as wireworms, represent one of the most widespread insect families, with nearly 10,000 described species [1,2,3]. Click beetles of the genus *Agriotes* are considered major pests of many economically important arable and vegetable crops across Europe and North America [4,5]. In Italy, over 200 species belonging to the genus *Agriotes* have been reported, with four species identified as serious crop pests: *A. sordidus* Illiger, *A. ustulatus* Schäller, *A. litigiosus* Rossi, and *A. brevis* Candèze [6,7,8]. *A. sordidus* has been reported as a major pest in western Germany [9] and France [10] and, together with *A. brevis*, represents one of the principal causes of severe damage to maize crops. *A. brevis* is also considered a major pest in several eastern European countries [7] and is present in western Europe [11]. *A. ustulatus* is associated with significant crop losses in central and eastern Europe [9,12,13,14,15], whereas *A. litigiosus* is recognized as a major pest in Greece and several eastern European regions [12,15,16,17,18].

Elaterids are characterized by a multi-year life cycle that varies among species [19,20,21], which is an important factor to consider when developing pest-control strategies. Their life cycle involves complete metamorphosis, with four main stages of development: egg, larva, pupa, and adult. Female click beetles lay eggs just under the moist soil surface [21,22]. Egg development is mainly related to temperature, requiring 13 days at 25 °C and 45 days at 15 °C. Larvae undergo multiple stages, typically from 8 to 13 instars, depending on temperature, soil moisture, and food availability, all of which affect the duration of their life cycle [19,21,23]. In spring, when soil temperatures increase, wireworms start actively feeding on the roots of both wild and cultivated plants, attracted by the CO_2_ and organic volatiles released by the roots [23,24]. In autumn, they burrow deep into the soil to overwinter and re-emerge in spring to resume their active feeding. Larval development in *A. ustulatus* and *A. sordidus* is absent or slow at temperatures below 9 °C [19,21]. The pupal stage lasts 13 days at 20 °C [19], with pupation occurring in late summer, late spring or early summer depending on the species. Adults emerge in early spring, just after temperatures reach 9–10 °C, and remain active until early autumn [21,25].

The polyphagous feeding habits of wireworms and their ability to dwell deep in the soil enable them to attack and feed on the seeds, seedlings, and emerging sprouts of various crops, including *Zea mays*, *Solanum lycopersicum*, *S. tuberosum*, *Daucus carota*, *Beta vulgaris*, and *Triticum aestivum*, leading to significant yield losses of up to 25% [25,26,27,28]. Tuber crops, such as *S. tuberosum*, are particularly vulnerable to high infestations of wireworms [29,30]. This leads to impaired plant growth and increased susceptibility to secondary infections by various diseases, which reduce crop quality [31].

Traditional practices to control elaterid populations have relied on the use of insecticides or insecticide-coated seeds [32,33]. The introduction of the European Directive 128/2009/EC resulted in the deregistration of various insecticides, leading to the re-emergence of the pest problem in several countries [34,35,36]. As opposed to relying on synthetic insecticides, the Integrated Pest Management (IPM) framework promotes the use of alternative methods, along with the integration of knowledge, monitoring, and predictions, to support decision-making for pest management [37]. Due to the variation in the life cycles and potential damage caused by different *Agriotes* species [38], a thorough understanding of their species composition, abundance, and emergence patterns in a given area is essential for developing effective IPM strategies.

In Italy, adult emergence times vary among *Agriotes* species: *A. brevis* emerges beginning in March, *A. sordidus* from late March to early April [21], *A. litigiosus* beginning in May [16], and *A. ustulatus* beginning in early June [19]. Moreover, *A. ustulatus* and *A. litigiosus* do not overwinter as adults; they live only a few days as adults, and they mate and lay eggs shortly after emerging. In contrast, *A. brevis* and *A. sordidus* can overwinter as adults, surviving for several months and laying eggs over an extended period [39]. Monitoring can be carried out using various methods, including sex-pheromone traps to capture adults. Several sex-pheromone traps have been developed to capture adult *Agriotes* species, including the Vernon beetle trap, Unitrap, baited pitfall trap, Vernon pitfall trap, Original YATLOR funnel trap, and YATLOR funnel trap [8,40]. The latter is suitable for monitoring both flying and crawling species throughout the season, mainly capturing adult males and a limited number of females [8]. However, most of the available data on *Agriotes* species come from northern Italy and other European regions [16,21], whereas information from central Italy remains scarce, despite increasing reports of wireworm damage in horticultural crops [8,26].

Furthermore, insect emergence patterns are strongly influenced by temperature and thermal accumulation [21,41], and climate change may shift phenological windows, potentially increasing crop vulnerability [28,41]. For this reason, updating regional data on species composition and emergence dynamics is crucial to refining monitoring tools and improving predictive models [41].

Another important aspect for improving pest management is the investigation of the heat accumulation required for the development of the different *Agriotes* species, as this parameter supports the prediction of adult emergence times and the optimal timing of monitoring and control actions [41]. Degree-day data are available for *A. sordidus* [21], but remain scarce for *A. brevis* and *A. litigiosus*. Also, elaterid damage has become increasingly significant in horticultural crops in central Italy, yet information on the local presence, abundance, and emergence dynamics of these pests is still limited [26,39]. To fill this gap, we provide information on the species composition and adult emergence patterns of *Agriotes* species in central Italy. Specifically, our aims were to: (i) assess the composition of *Agriotes* species, (ii) investigate their relative population abundance, and (iii) investigate the adult emergence patterns in relation to heat accumulation to provide new insights for the development of knowledge-based and sustainable IPM strategies.

## 2. Materials and Methods

### 2.1. Study Sites

**Figure 1:**
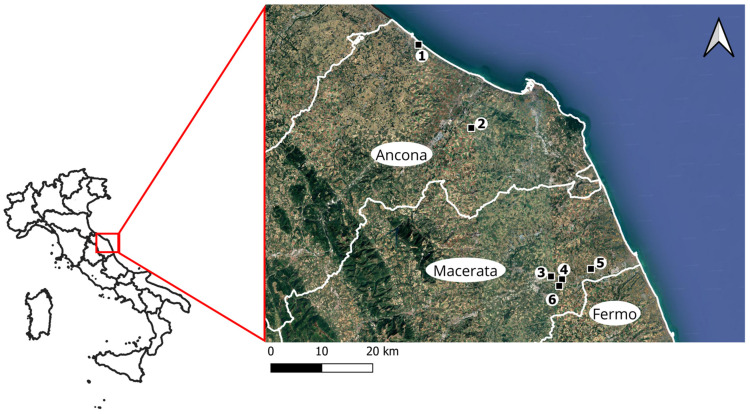
Location of the study fields monitored for *A. brevis*, *A. sordidus*, *A. litigiosus*, and *A. ustulatus* populations in the Marche region (central Italy) during 2024–2025.

Study sites were located in coastal and hilly areas of the Marche region. These sites were selected based on farmer and phytosanitary service reports of wireworm damage to crops. In 2024 and 2025, six sites from different areas of the Marche region were chosen to monitor adults of the click-beetle species (Figure 1). Details about the study fields are reported in Table 1. Soil characteristics, obtained from laboratory analyses conducted by an external specialized consultancy, are presented in Table 2. Agronomic details provided by the agronomists of the participating farms are listed in Table 3. Some study sites consisted of heterogeneous cropping systems, where multiple crop species were cultivated simultaneously within the same field or in adjacent plots.

### 2.2. Adult Sampling

**Figure 2:**
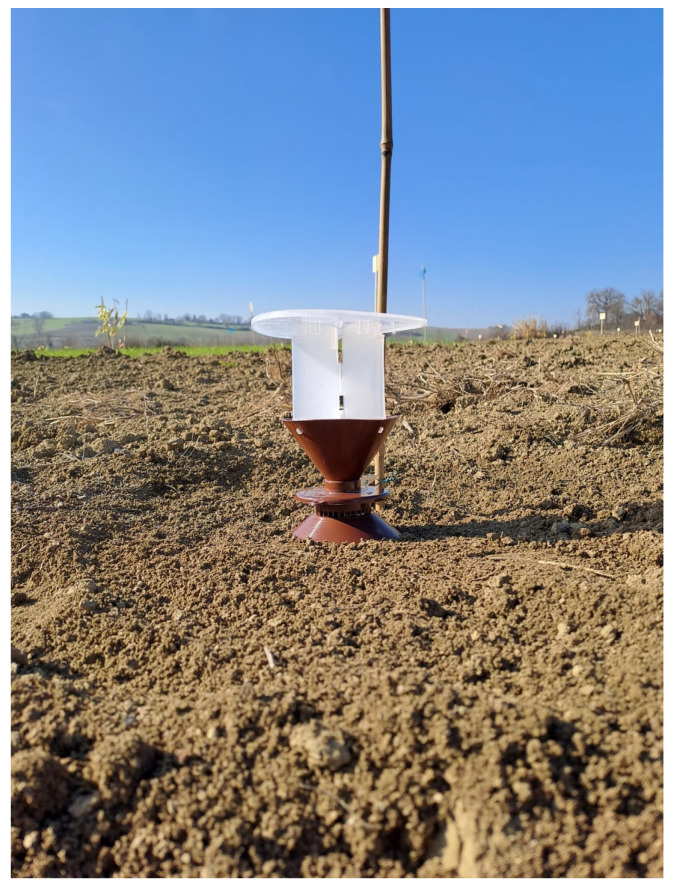
YATLORf trap used for monitoring click-beetle adults.

YATLORf traps, produced by ISAGRO—Italian Creative for Plant Health (Adria, Rovigo, Veneto, Italy), were used for monitoring adults (Figure 2). This trap consists of a white upper section designed to hold the pheromone, a brown funnel-shaped edge with a scale to capture the adults, and a white lower section for anchoring the trap in the soil. In the field, these traps were positioned with the white lower section buried facing downwards and the brown edge placed 1–2 cm below the soil surface [42]. The traps were baited with commercial sex-pheromone lure dispensers purchased from the Hungarian company CSALOMON^®^ (Plant Protection Institute, Budapest, Hungary). The sex-pheromone dispenser was placed inside the narrowest part of the funnel, sealed, and positioned upside down. This setup is suitable for all species and field conditions [42]. The pheromones were specific to *Agriotes brevis*, with geranyl butanoate + (E,E)-farnesyl butanoate in a 1:1 ratio (15 + 15 mg); *Agriotes sordidus*, with geranyl hexanoate (30 mg; *Agriotes litigiosus*: geranyl isovalerate (50 mg); and *Agriotes ustulatus*, with (E,E)-farnesyl acetate (50 mg) [43,44]. At each site, 12 traps were arranged into 3 blocks. Each block contained four traps; each trap was baited with the attractant specific to each of the four *Agriotes* species. All traps were placed along the borders of the study field. Adjacent traps within each block were set 20 m apart. The traps were inspected weekly, and the baits were replaced every four weeks. The area surrounding the traps was consistently cleaned of any wild vegetation. Specimens from each trap were collected into individual 80 mL plastic rearing boxes, labelled with the date and trap location, and transported to the laboratory for species identification, sex determination, and counting. Adult monitoring was carried out from January 2024 to September 2025. Identification of adult specimens was carried out using the dichotomous key developed by Platia (1994) [6].

### 2.3. Data Elaboration and Analysis

Differences in mean adult abundance among species per year, and within species among years were evaluated using Kruskal–Wallis tests. Analyses were performed both across all sites combined and separately for each site. Post hoc pairwise comparisons were conducted using Dunn’s test with Bonferroni correction, with significance set at *p* < 0.05.

Adult trap catches of each species were pooled across all locations to calculate the overall cumulative percentage of emergence. This was calculated as the relative cumulative adult abundance for each sampling date, divided by the total number of adults collected over the entire flight period. Site-specific air temperature data were obtained from the nearest weather station, located less than 5 km from each site, through the Regional Meteorological-Hydro-Pluviometric Information System of the Marche Region http://app.protezionecivile.marche.it/sol/indexjs.sol?lang=it (accessed on 19 September 2025). Hourly air-temperature data were used to calculate cumulative degree-days (DD) based on the formula

$$DD=\sum_{i=1}^{n}\frac{max\left[0,\left(T_{i}-T_{0}\right)\right]}{24}$$

where n is the number of hours in one year (from 1 January to 31 December), $T_{i}$ is the air temperature at hour i, and $T_{0}$ represents the lower developmental threshold temperature. In the present study $T_{0}$ was set to 9 °C following the available information provided by Furlan [21]. For each site and year, we estimated the cumulative DD required to reach the 1st (onset of flight), 50th, and 99th (end of flight) percentiles of the cumulative percentage of adult emergence. All analyses were conducted in R (version 4.3.1).

## 3. Results

### 3.1. Species Composition and Abundance

**Figure 3:**
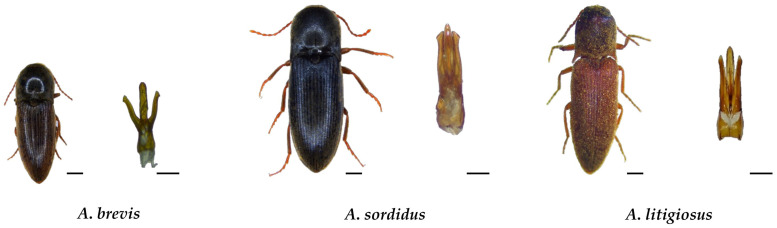
Pictures of the *Agriotes* species captured and identified in the Marche region. From left to right, adult stage (scale bar: 1 mm) and aedeagus (scale bar: 0.25 mm).

**Figure 4:**
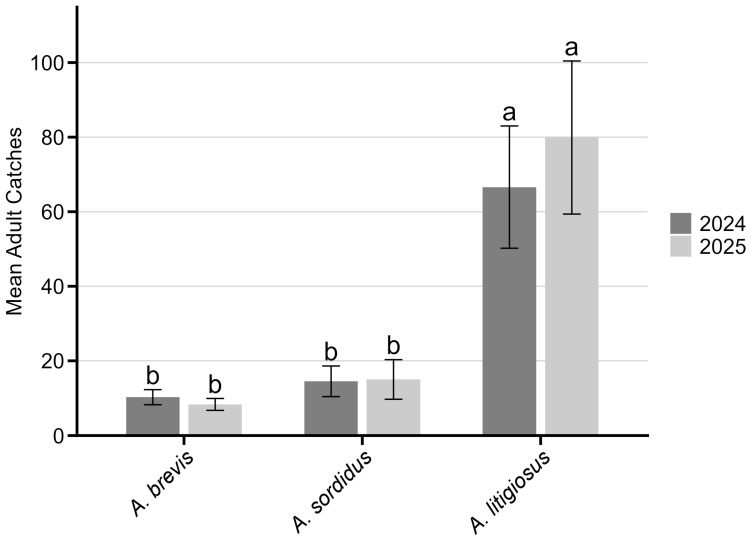
Mean (±95% confidence interval) adult catches of *Agriotes brevis*, *A. sordidus* and *A. litigiosus* at monitored sites in Marche region in 2024 and 2025. Different letters indicate significant differences among species within each year (Dunn post hoc test, *p* < 0.05).

In the monitored areas, adult monitoring activities detected the presence of *A. litigiosus* (with 11,189 individuals collected), *A. sordidus* (3998 individuals), and *A. brevis* (3337 individuals) (Figure 3). No specimens of *A. ustulatus* were collected during the adult monitoring activities. Considering the overall number of adult catches, significant differences were observed between the three species in 2024 (KW chi-squared = 10.89, df = 2, *p* < 0.005) and 2025 (KW chi-squared = 11.94, df = 2, *p* < 0.005). In both years, the post hoc Dunn test indicated that *A. litigiosus* had significantly higher catches compared to *A. brevis* (*p* < 0.05) and *A. sordidus* (*p* < 0.01). No significant difference was found between *A. brevis* and *A. sordidus* (Figure 4). No significant differences were observed for any species across years.

**Figure 5:**
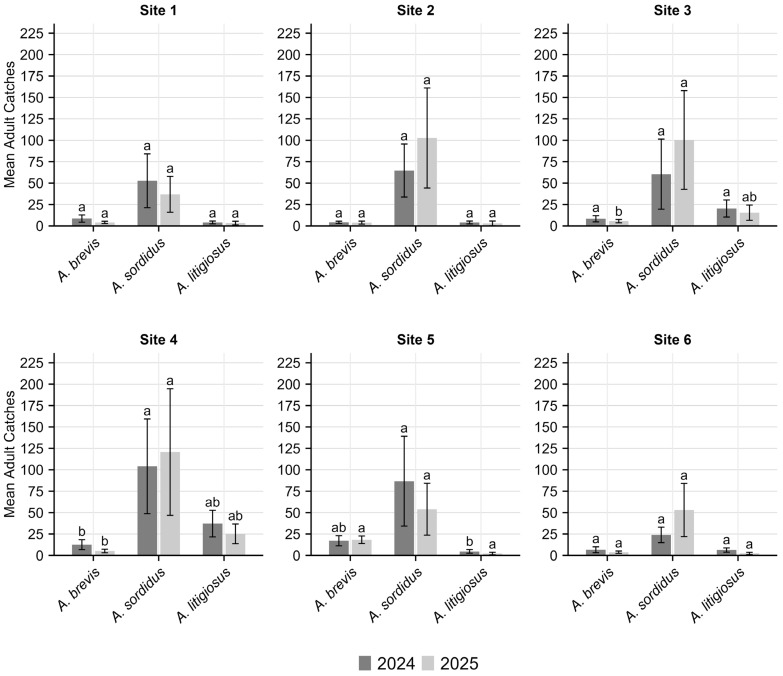
Site-specific mean (±95% confidence interval) adult catches of *Agriotes brevis*, *A. sordidus* and *A. litigiosus* in the Marche region in 2024 and 2025. Different letters indicate significant differences among species within each year (Dunn post hoc test, *p* < 0.05).

Among the total adults captured, a total of 10, 184, and 73 females were recorded for *A. litigiosus*, *A. sordidus*, and *A. brevis*, respectively. Significant differences in adult trap catches among species were observed for site 4 and site 5 in 2024, and for site 3 and site 4 in 2025 (*p* < 0.05) (Figure 5). The post hoc Dunn test showed significantly lower catches of *A. brevis* compared to *A. litigiosus* in site 4 in 2024 (*p* < 0.05), and in sites 3 and 4 in 2025 (*p* < 0.05). Catches of *A. sordidus* were significantly lower compared to *A. litigiosus* in site 5 in year 2025 (*p* < 0.05).

### 3.2. Adult Population Dynamics and Phenology

**Figure 6:**
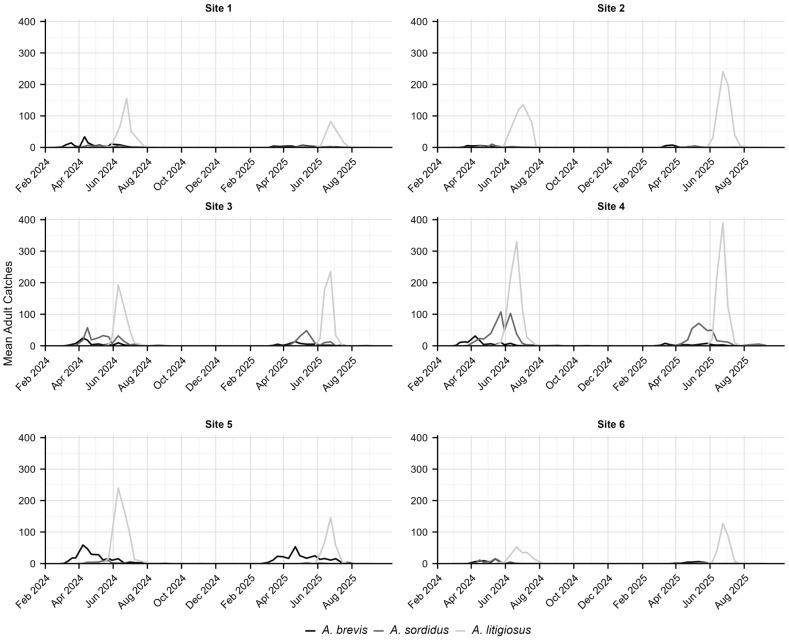
Site-specific temporal dynamics of *Agriotes brevis*, *A. sordidus* and *A. litigiosus* in the Marche region in 2024 and 2025.

The site-specific population dynamics are shown in Figure 6. In the monitored sites, the first individuals of *A. brevis* emerged at the beginning of March, followed by *A. sordidus* (from late March to early April) and *A. litigiosus* (from late May to early June). The flight periods of *A. brevis* and *A. sordidus* were relatively longer (approximately 120–170 days) compared to *A. litigiosus* (around 60–70 days). The highest number of adult catches during peak periods was *A. litigiosus* (with more than 300 individuals per trap per week), followed by *A. brevis* (ca. 40–100 individuals per trap per week) and *A. sordidus* (ca. 10–60 individuals per trap per week).

**Figure 7:**
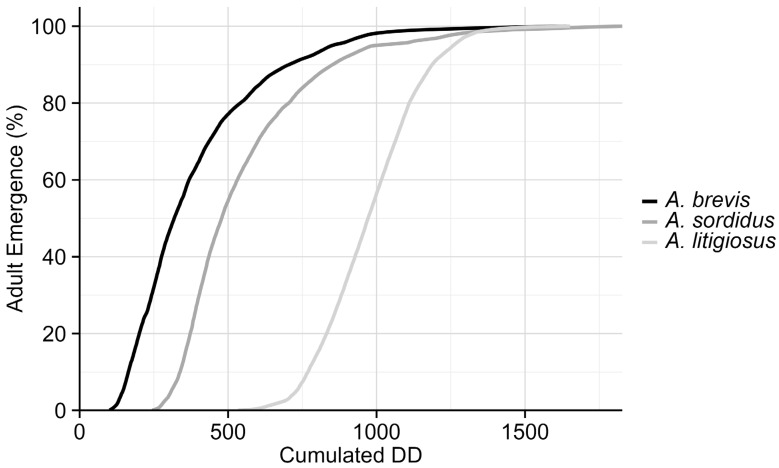
Mean cumulated emergence of adults (%) in relation to mean cumulated degree-days (DD) for *Agriotes brevis*, *A. sordidus*, and *A. litigiosus* at monitored sites in the Marche region, averaged across 2024 and 2025.

**Figure 8:**
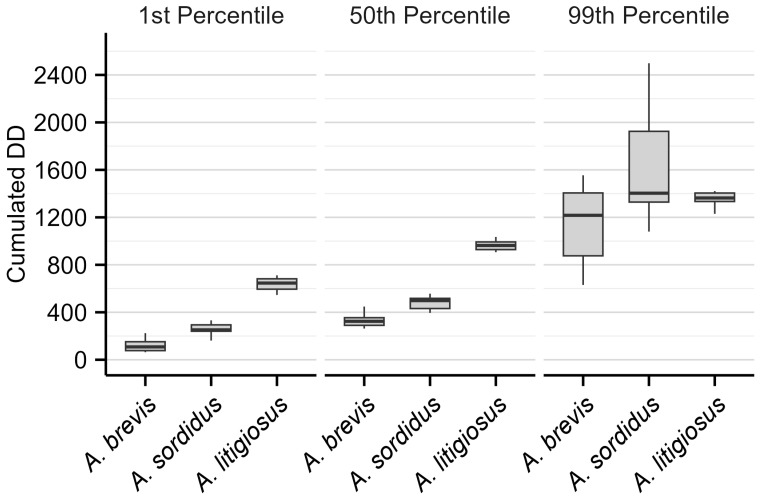
Boxplots showing the cumulated degree-days (DD) required to reach the 1st, 50th, and 99th percentile of adult emergence for *Agriotes brevis*, *A. sordidus*, and *A. litigiosus*. Percentiles were calculated by pooling data across 2024 and 2025. The solid line represents the median. Lower and upper hinges correspond to the first and third quartiles. The whisker extends from the hinge to the smallest/largest value, no further than 1.5 times the inter-quartile range.

Figure 7 shows the cumulative adult emergence in relation to cumulative DD averaged across the two monitoring years (2024 and 2025). The graph clearly illustrates that *A. brevis* requires the least heat accumulation for emergence, followed by *A. sordidus* and *A. litigiosus*. This is further confirmed by Figure 8, which indicates that the onset of adult emergence requires 108 DD for *A. brevis*, followed by *A. sordidus* (252 DD) and *A. litigiosus* (646 DD). The 50th percentile of adult emergence showed the same pattern with 324 DD required for *A. brevis*, 498 DD for *A. sordidus*, and 963 DD for *A. litigiosus*. The flight period for *A. brevis* concluded after 1217 DD, followed by *A. litigiosus* (1363 DD) and *A. sordidus* (1403 DD). Overall, *A. sordidus* required the greatest heat accumulation (1152 DD) to complete its flight, comparable to what is observed for *A. brevis* (1109 DD), and lastly *A. litigiosus* with 717 DD. These findings confirm the shorter temporal and thermal presence window of *A. litigiosus* compared to the other species.

## 4. Discussion

In this study, we detected the presence of *A. brevis*, *A. sordidus*, and *A. litigiosus* in the monitored areas in central Italy. The highest catches were recorded for *A. litigiosus*, followed by *A. sordidus* and *A. brevis*. These results are in accordance with Platia (1994) [6], and Furlan et al. (2001 and 2021) [18,39], who observed the presence of the same species across some regions of northern Italy, such as Veneto, Emilia-Romagna, Lombardia, and Piemonte. The absence of *A. ustulatus* aligns with its distribution, which is restricted to northeastern Italy [45]. Higher catches of *A. litigiosus* can be attributed to the presence of mainly clayey soils in the investigated sites, which are more suitable for the pest, as reported in Veneto and Emilia-Romagna by Furlan et al. (2000) [45]. In contrast, *A. sordidus* is known for its adaptability to lighter soils, while *A. brevis* is better suited to sandy soils [45]. Given the high trap catch rates, our study indicates the potential of *A. litigiosus* to be a serious threat to crops in central Italy, as previously reported for Greece [28] and Eastern European countries [16]. Main potential damages in central Italy are reported for potato, but there are also risks for maize, lettuce, spinach, and fennel, especially in cases of high wireworm population density [7]. The pheromone trap catches reflect not only species abundance but also behaviour, such as flight propensity and plume perception.

Any comparative evaluation of population densities based on trap catches should consider the different behaviours of the investigated species. Trials conducted by Furlan et al. (2021) [8] showed that *A. brevis*, a predominantly crawling species, had the lowest number of catches, approximately two to four times lower than *A. litigiosus*. The latter has a strong tendency to fly, and it is likely that this greater flight propensity increases the probability of beetles entering the area where pheromone plumes can be perceived, likely partially explaining the higher catches of *A. litigiosus* in the monitored areas [42]. Sex-pheromone traps captured both males and females, with a higher number of males than females at all monitored sites. This finding confirms previous findings reported for *A. brevis* [46] and *A. sordidus* [44].

In relation to adult emergence patterns, we observed the adult emergence of *A. brevis* at the beginning of March, which is earlier than the findings of Landl et al. (2010) [47], who reported the first *A. brevis* emergence in Austria at the end of April during 2004–2005, and Subchev et al. (2010) [5], who recorded the first catches in Bulgaria during the third week of May 2005. For *A. sordidus*, adult emergence was observed from the end of March to the first decade of April, with a peak from the end of April to May. This aligns with findings by Furlan (2004) [21], who studied the biology of *A. sordidus* over 10 years in northern and southern Italy. We observed the adult emergence of *A. litigiosus* at the end of May, with an emergence peak in June, consistent with the results of Karabatsas et al. (2001) [16], who monitored *Agriotes* species in Greece over four years from 1998 through 2001. All observations from experiments and literature indicate that the activity of *Agriotes* adults varies across different locations [14,48,49]; nevertheless, it remains similar when climatic conditions are comparable [47], highlighting the importance of weather variables in affecting the life-cycle of these pests. In this context, climate warming may advance the onset of adult flight activity and/or extend its duration, resulting in earlier and more prolonged periods of adult presence in the field. As a result, the temporal window during which adult activity overlaps with crop susceptibility may increase, thereby raising the risk of damage. This underlines the importance of developing predictive degree-day models adapted to Mediterranean conditions.

The phenological analysis, linking the cumulated emergence of *Agriotes* adults to cumulative DD, showed that *A. brevis* required the fewest DD for the beginning of adult activity, followed by *A. sordidus* and *A. litigiosus*. This pattern was consistent for the 50th percentile of emergence. *Agriotes brevis* also completed its flight first, followed by *A. litigiosus* and *A. sordidus*. The two species that can overwinter as adults (*A. sordidus* and *A. brevis*) showed a prolonged flight period, while *A. litigiosus*, which overwinters exclusively as larvae, had a shorter but more abundant flight window. The shorter, yet prominent, presence of *A. litigiosus* has significant management implications. The relatively high abundances reached by the pest might cause significant damage to crops; however, its concentrated activity period makes it potentially easier to target the optimal timing to implement management actions against adults and/or newly hatched larvae. In contrast, the longer flight periods of *A. brevis* and *A. sordidus* result in a requirement for extended monitoring and control efforts to be carefully implemented throughout the growing season. From an IPM perspective, these differences indicate that *A. litigiosus* may be more manageable through targeted, short-term interventions, whereas *A. brevis* and *A. sordidus* require prolonged surveillance and staggered measures.

Although the present study is based on field monitoring conducted over a relatively limited temporal and spatial scale, it provides original and scientifically relevant ecological information that was previously unavailable for the investigated *Agriotes* species, and which can be used to optimize IPM strategies. In particular, no published data were available on the relative abundances of the target species or on the species-specific adult emergence patterns expressed as cumulative degree-days (DD).

Degree-day analyses rely on well-established physiological principles governing insect development [50] and provide a standardized framework that facilitates phenological comparisons across species, years, and regions [51,52]. While absolute cumulative DD values should be interpreted with caution, especially in the absence of species-specific and stage-specific activity thresholds, the relative timing and shape of emergence curves remain robust descriptors of phenological differences among species. Given the lack of species-specific adult activity thresholds in the literature, we adopted a lower developmental threshold of 9 °C, commonly used to describe larval development and the completion of pre-imaginal stages in *Agriotes sordidus* [21], as a pragmatic and conservative reference value. This threshold was applied consistently across species and years, ensuring internal coherence and enabling robust relative comparisons of emergence timing, flight duration, and variability.

Importantly, this study aims to use the best available information to characterize field emergence dynamics and their implications for population ecology and pest management. In this regard, field-based phenological studies should be viewed as complementary to laboratory or controlled-condition experiments, which are essential for elucidating mechanistic processes and identifying species- and stage-specific activity thresholds.

The information we have provided complements the existing knowledge about the life cycle of *A. sordidus*, which has been extensively studied by Furlan (2004) [21], and offers new insights into species whose life cycles are less well known (*A. brevis* and *A. litigiosus*). When compared with studies conducted in other European regions, such as Austria [47], Greece [16], and Bulgaria [5], our results indicate both shared trends and regional peculiarities, reinforcing the importance of local monitoring. A more comprehensive analysis of the flight patterns of these species will require several consecutive years of data to fully capture their life cycles and to identify potential differences or recurring patterns in their flight dynamics. Such long-term datasets will also be fundamental to assessing phenological shifts driven by climate change and to integrating these dynamics into decision-support systems for farmers.

## 5. Conclusions

Effective management of *Agriotes* species requires a thorough understanding of their species composition, life cycles, and emergence patterns. In this study, we confirmed the presence of *A. litigiosus, A. sordidus,* and *A. brevis* in central Italy, with *A. litigiosus* being the most abundant species. Differences in adult emergence timing and flight periods among species underscore the need for species-specific monitoring and tailored management strategies. Our findings may contribute to the development of knowledge-based pest management approaches by providing detailed information on the species composition, abundance, and phenology of *Agriotes* spp. in central Italy. The identification of the dominant species (*A. litigiosus*) allows management efforts to be primarily focused on this species. The characterization of species-specific emergence patterns enables improved timing of monitoring and control actions, particularly targeting periods of peak adult activity and early larval stages. The results of this work support the optimization of IPM strategies by enabling more targeted interventions for species with short flight periods, such as *A. litigiosus*, while highlighting the need for prolonged monitoring and staggered control measures for species with extended flight activity, such as *A. brevis* and *A. sordidus*. Overall, management actions guided by these results may lead to a more rational and reduced use of chemical treatments, improving control efficacy while minimizing risks to the environment and non-target organisms. However, long-term studies are still needed to refine predictive models and assess the influence of environmental factors on *Agriotes* populations, ultimately enhancing IPM strategies in Mediterranean agricultural systems.

## Figures and Tables

**Table 1:** Study fields monitored for *A. brevis*, *A. sordidus*, *A. litigiosus*, and *A. ustulatus* in the Marche region (central Italy) during 2024–2025. The table reports site location, coordinates, crop cultivated during the monitoring years, and crop from the previous year.

Site, Location, Province	Dec_Lat, Dec_Lon	Crop in the Monitoring Years	Crop in the Previous Year
1 Senigallia, Ancona	43.696, 13.242	*Spinacia oleracea* *Beta vulgaris* *Foeniculum vulgare* *Capsicum annuum* *Lactuca sativa* *Cucurbita moschata* *Cucumis melo* *Citrullus lanatus*	*Brassica oleracea* *Cichoriums intybus* *Zea mays* *Solanum lycopersicum*
2 Agugliano, Ancona	43.545, 13.360	*Zea mays* *Vicia faba* *Helianthus annuus* *Triticum aestivum*	*V. faba* *H. annuus* *T. aestivum* *Z. mays*
3 Montecosaro, Macerata	43.284, 13.631	*L. sativa* *F. vulgare* *Pisum sativum* *Phaseolus* *Z. mays* *C. moschata*	*Triticum durum* *B. oleracea* *S. oleracea* *C. intybus*
4 Trodica di Morrovalle, Macerata	43.275, 13.534	*L. sativa* *Sorghum bicolor*	*T. durum*
5 Trodica di Morrovalle, Macerata	43.2688, 13.560	*L. sativa* *Z. mays* *S. oleracea*	*C. intybus* *S. oleracea*
6 Villa San Filippo, Macerata	43.257, 13.552	*C. annuum* *H. annuus*	*T. durum*

**Table 2:** Soil characteristics of the study fields monitored for *A. brevis*, *A. sordidus*, *A. litigiosus*, and *A. ustulatus* in the Marche region (central Italy) during 2024–2025. The soil texture triangle was classified using the USDA soil texture triangle; values of organic matter, C/N ratio, and pH were also recorded.

Site and Soil Texture	% (Silt, Clay, Sand)	g/kg (Organic Matter)	C/N Ratio	pH
1 Clay loam	23.1, 38.3, 38.6	22.4	19.9	8.1
2 Clay	50.3, 44.2, 5.5	27.5	16.2	8.3
3 Clay loam	22.9, 37.9, 39.2	23.9	20.4	8.13
4 Sandy clay Loam	17.2, 34.6, 48.2	19.1	16.3	8.21
5 Clay loam	23.5, 31.2, 45.3	20.4	17.3	8.24
6 Clay	16.0, 50.0, 34.0	20.7	8	7.3

**Table 3:** Agronomic information on the study fields monitored for *A. brevis*, *A. sordidus*, *A. litigiosus*, and *A. ustulatus* in the Marche region (central Italy) during 2024–2025. Details include farm type, soil tillage practices, plant protection treatments, irrigation, and fertilizer applications.

Site and Farm Type	Soil Tillage Practices	Plant Protection Treatments Applied	Irrigation Practices	(%) Fertilizers Applied (Micronutrients and Macronutrients)
1 Organic	Shredding Subsoiling Tilling Weeding	Pyrethroids Neem Oil Orange Essential Oil Copper Sulfur Potassium Bicarbonate	Micro-irrigation	3 P_2_O_5_, 8 P_2_O_5_ 2 N, 6 N 2 K_2_O, 15 K_2_O 10 CaO 1 MgO, 2 MgO 7 SO_3_ 0.03 Zn
2 Conventional	Ploughing Minimum tillage	Glyphosate Chlorantraniliprole Deltamethrin *Bacillus thuringiensis*	None	27 NH_4_NO_3_, 46 NH_4_NO_3_ 18 N, 46 N 20 P_2_O_5_
3 Conventional	Ploughing Refinement Ridging	Pendimethalin Difeconazole-Fluxapyroxad Lambda-Cyhalothrin Cyprodinil-Fludioxonil	Reel irrigator and micro-irrigation	34 NH_4_NO_3_ 12 N, 6 N, 46 N 12 P 17 K 2 MgO 20 SO_3_ 0.02 B 0.01 Zn
4 Conventional	Ploughing Refinement	Pendimethalin Boscalid–Pyraclorobin Spirotetramat Chlorantraniliprole Difeconazole–Fluxapyroxad Tebufenzoide Lambda-Cyhalothrin Copper Cyprodinil-Fludioxonil	None	46 N
5 Conventional	Ploughing Refinement Ridging	Pendimethalin Boscalid–Pyraclorobin Spirotetramat Chlorantraniliprole Difeconazole–Fluxapyroxad Tebufenzoide Lambda-Cyhalothrin Copper Cyprodinil-Fludioxonil	Reel irrigator and micro-irrigation	4 N, 25 N, 12 N 4 P, 12 P, 15 P 4 K, 17 K
6 Conventional	Ploughing Refinement Subsoiling	Ferric Phosphate S-metolachlor	None	19 P_2_O_5_, 30 P_2_O_5_ 10 N, 24 N

## Data Availability

The original contributions presented in this study are included in the article. Further inquiries can be directed to the corresponding author.
